# Discovery of Poison in a Body after Eight Year’s Interment, at Westbury, Wilts

**Published:** 1849-11

**Authors:** 


*Discovery of Poison in a Body after Eight Years' Interment, at Westbury, Wilts.*

In consequence of the recent inquest here, at which a verdict of "Wilful murder" was returned against Rebecca Smith, for procuring the death of her infant child by the administration of poison, it was deemed advisable to exhume some of the bodies of the nine other children, who have all died in infancy. Accordingly, on the 11th inst., two bodies were disinterred from the burying-ground of the baptist chapel, at Bratton, in this parish, under the superintendence of Mr. Shorland, surgeon of Westbury, by whom the remains were taken to Mr. Herapath, of Bristol, for analyzation. A coroner's inquest sat on that day, which was adjourned till this day, after taking evidence of the identity of the exhumed bodies.

At the resumed sitting, Mr. Shorland described the state in which he forwarded the bodies to Mr. Herapath; after which that gentleman gave the following curious and important evidence:—"On the 12th inst., on my return from Exeter, I found at my laboratory a large, square, shallow box, on which the cover was sealed down with a crest similar to that on Mr.

Shorland's seal. This seal was perfect. The box was divided into three compartments by two divisions, in one of which was a portion of soil tied up in a handkerchief. In the next compartment I found a mass of earth and the remains of a coffin, exceedingly decomposed, and penetrated in all directions by the roots of a tree. There was a label, in Mr. Shorland's handwriting, on the top of this, to this effect: 'Sarah Smith, born July 18, 1841; died August 7, 1841, aged 20 days.' Upon carefully removing portions of the soil, I found the remains of an infant, evidently very young, as there were no teeth in the sockets of the jaw, with the exception of one tooth-bud on the front of the lower jaw. The texture of the body was entirely gone, and the bones were all separated from each other. I took some of the bones, and subjected them to analysis, when I found in them traces of arsenic. I then took some of the black mould from the interior of the skull, and in that I also found traces of arsenic. I then sought for some of the black mould between the ribs, and nearer the region of the stomach, and there I found arsenic in greater quantity,—specimens of which I produce." Mr. Herapath then exibited tubes containing arsenious acid, metallic arsenic, Scheele's green, and orpiment, produced by various tests, and continued:—"This, I believe, is the first instance on record of arsenic being discovered after an interment of eight years, and I wish it to be circulated throughout the country that years have no effect in removing traces of arsenic. In the third compartment I found, also, the remains of an infant, with a label, in Mr. Shorland's handwriting, as follows: 'Edward Smith, born June 14, 1844; died June 29, 1844, aged 15 days.' This body and coffin were nearly in the same state as the others; the bones below the knees were wanting. The roots of trees as large as my little finger had passed through the head and skeleton, and had followed the bones in all directions. Treating this skeleton as I did the other, I found arsenic in the bones, in the black mould under the head, and a greater quantity in the black mould under the ribs. I produce specimens of metallic arsenic, and the other tests which are even more distinct than those in the last case: this is after an interment of five years and one month."

The coroner.—From the statement you have made, and from your analysis, have you any doubt that arsenic was administered during life?

Mr. Herapath.—I have never found arsenic in a body which was in a natural state; and I mention this to correct the ridiculous notions which have gone abroad, owing to some sayings which have been attributed to the French chemists. Raspail, for instance, is reported to have said that he could produce arsenic from the legs of chairs; and Orfila, that he could do so from the common soil. I have made experiments on hundreds of bodies of human beings and brutes, but have never discovered arsenic unless it had been administered medicinally, or for a criminal purpose. I have also made many experiments on soils, and I believe the statement of Orfila to be a mistaken one. My opinion is, that arsenic was administered to both these children during life, and that it was the cause of death; it existed in too great a quantity to have been administored for a medicinal purpose.

The jury, without hesitation, returned a verdict "That the deceased children died from the effects of arsenic, but how or by whom administered there is no evidence to show."—*London Times.*

